# Supplementary material for: The persistence potential of transferable plasmids
Source: Nat Commun. 2020 Nov 4;11:5589. doi: 10.1038/s41467-020-19368-7 (PMC7642394; doi:10.1038/s41467-020-19368-7)
Supplement: Supplementary file 1 — Supplementary Information [file 41467_2020_19368_MOESM1_ESM.pdf]

Supplementary Information for

The Persistence Potential of Transferable Plasmids

Teng Wang, Lingchong You*

*Correspondence to: you@duke.edu

This file includes:

Supplementary Methods
Supplementary Text
Supplementary Figures 1 to 5
Supplementary Tables 1 to 16

Section 1: Supplementary methods

Strains and plasmids

The compositions of the eight engineered communities are shown in Supplementary Table 1. *E. coli* strain MG1655 without fluorescence markers was denoted as strain X. *E.coli* strain DA26735 with chromosomal BFP and chloramphenicol resistance (Cm^R) was denoted as strain B, and *E.coli* strain DA32838 with chromosomal dTomato and Cm^R was denoted as strain R. All three strains carry helper F plasmid F_{HR} that expresses tetracycline resistance (Tet^R). F_{HR} is not transmissible but encodes the conjugation machinery that mobilize plasmid K[1]. Plasmid K expresses GFP under the control of a strong constitutive PR promoter and expresses kanamycin resistance (Kan^R). Plasmid K also carries *oriT*, so it can be transferred through conjugation.

The multi-plasmid community was composed of two *E. coli* strains, MG1655 and DH5α. These strains were distinguished from each other via blue-white screening on X-gal plates, where MG1655 and DH5α colonies were blue and white, respectively. These communities transferred five conjugative plasmids: F' (lncF, Tet^R), PCU1 (lncN, Amp^R), R388 (lncW, Tm^R), R6K (lncX, $Strp^R$), and RP4 (lncP, Kan^R). These five plasmids are compatible with each other and carry different antibiotic resistance markers. The plasmids were distinguished from each other via selective plating.

Supplementary Table 1 The strain compositions and plasmids in the eight engineered communities

communities	strains	plasmids
1	X (MG1655+F_{HR})	K (Kan^R, GFP)
2	B (DA26735+F_{HR})	
3	R (DA32838+F_{HR})	
4	X, B	
5	X, R	
6	B, R	
7	X, B, R	
8	MG1655, DH5*α*	F' (lncF, Tet^R), PCU1 (lncN, Amp^R),R388 (lncW, Tm^R), R6K (lncX, $Strp^R$), and RP4 (lncP, Kan^R)

Section 2: Model and theoretical Analysis

2.1 Model development of plasmid-centric framework (PCF)

2.1.1 The matrix form of the plasmid-centric framework

Using vectors and matrices, our plasmid-centric framework can be summarized into two equations

$$\frac{d\mathbf{S}}{dt} = \mathbf{A} \circ \mathbf{U_S} \circ \mathbf{S} \circ \mathbf{C} - D \cdot \boldsymbol{S} \quad (1)$$

$$\frac{d\mathbf{P}}{dt} = \mathbf{B} \circ \mathbf{U_P} \circ \mathbf{P} \circ \bar{\mathbf{C}} + (\bar{\mathbf{S}} - \mathbf{P}) \circ \mathbf{H} \circ \bar{\mathbf{P}} - D \cdot \mathbf{P} - \mathbf{K} \circ \mathbf{P}. \quad (2)$$

Here, we use communities of two species and two plasmids to illustrate the formations of each term in the equations. The formulations of communities with higher numbers of species and plasmids can be obtained in the same way. In the equations, '∘' is the Hadamard product (i.e., element-wise multiplication between two vectors or between two matrices). **S** and **P** represent the vectors or matrices of the abundances of species and plasmids, respectively:

$$\mathbf{S} = \begin{bmatrix} s_1 \\ s_2 \end{bmatrix}, \mathbf{P} = \begin{bmatrix} p_{11} & p_{12} \\ p_{21} & p_{22} \end{bmatrix}.$$

$\mathbf{A}$, $\mathbf{U_S}$ and $\mathbf{C}$ are the vector forms of α_i, μ_i and c_i, respectively:

$$\mathbf{A} = \begin{bmatrix} \alpha_1 \\ \alpha_2 \end{bmatrix}, \mathbf{U_S} = \begin{bmatrix} \mu_1 \\ \mu_2 \end{bmatrix}, \mathbf{C} = \begin{bmatrix} c_1 \\ c_2 \end{bmatrix}.$$

$\mathbf{B}$, $\mathbf{U_P}$ and $\bar{\mathbf{C}}$ are the matrix forms of β_{ij}, μ_{ij} and c_i, respectively:

$$\mathbf{B} = \begin{bmatrix} \beta_{11} & \beta_{12} \\ \beta_{21} & \beta_{22} \end{bmatrix}, \mathbf{U_P} = \begin{bmatrix} \mu_{11} & \mu_{12} \\ \mu_{21} & \mu_{22} \end{bmatrix}, \bar{\mathbf{C}} = \begin{bmatrix} c_1 & c_1 \\ c_2 & c_2 \end{bmatrix}.$$

$\bar{\mathbf{S}}$ is the expanded matrix of the vector **S**,

$$\bar{\mathbf{S}} = \begin{bmatrix} s_1 & s_1 \\ s_2 & s_2 \end{bmatrix}.$$

H contains the horizontal transfer rates, with the form

$$\mathbf{H} = \begin{bmatrix} [\eta_{111} & \eta_{121}] & [\eta_{211} & \eta_{221}] \\ [\eta_{112} & \eta_{122}] & [\eta_{212} & \eta_{222}] \end{bmatrix},$$

where η_{jki} is the conjugation rate constant of plasmid j from species k to species j. $\overline{\mathbf{P}}$ is expressed as

$$\overline{\mathbf{P}} = \begin{bmatrix} \begin{bmatrix} p_{11} \\ p_{21} \end{bmatrix} & \begin{bmatrix} p_{12} \\ p_{22} \end{bmatrix} \\ \begin{bmatrix} p_{11} \\ p_{21} \end{bmatrix} & \begin{bmatrix} p_{12} \\ p_{22} \end{bmatrix} \end{bmatrix}.$$

K contains the plasmid loss rates

$$\mathbf{K} = \begin{bmatrix} \kappa_{11} & \kappa_{12} \\ \kappa_{21} & \kappa_{22} \end{bmatrix}.$$

κ_{ij} represents the loss rate of plasmid j in species i.

2.1.2 Generalization of plasmid-centric framework

The basic principles of the PCF have been discussed in Method. Here, we will generalize the framework to accommodate plasmid incompatibility. Let G_l represent the incompatibility groups ($l = 1,2, \dots, t$, and $t \leq n$) and Z_j represent the map from plasmid index j to the index of its incompatibility group l. For instance, for plasmids j and k, if $Z_j = Z_k$, these two plasmids belong to the same incompatibility group and will not coexist stably in the same host cell. Therefore, the modified ODEs for p_{ij} become

$$\frac{dp_{ij}}{dt} = \beta_{ij}\mu_{ij}p_{ij}c_i + \left(s_i - \sum_{\{h:\, Z_h = Z_j\}} p_{ih}\right)\sum_{k=1}^{m} \eta_{jki}p_{kj} - (\kappa_{ij} + D)p_{ij} \qquad (3)$$

$$\beta_{ij} = \frac{s_i(1 + \lambda_{ij})}{s_i(1 + \lambda_{ij}) + \sum_{\{h:\, Z_h \neq Z_j\}}(p_{ih}\lambda_{ih})}. \qquad (4)$$

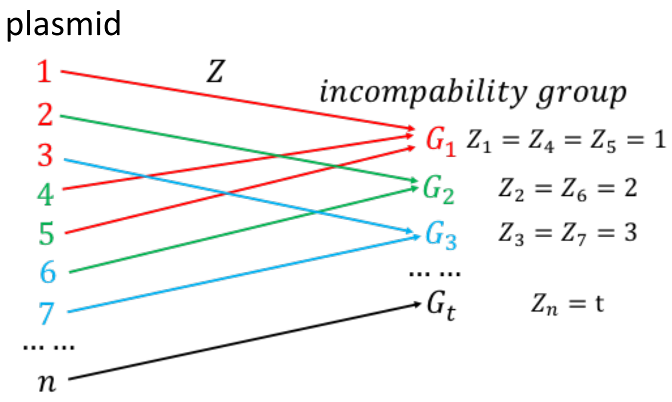

An example of a community containing 200 species and two incompatible plasmids is shown in Supplementary Fig. 1c.

Next, let's consider the general formulation of the carrying capacities c_i. We take niche partitioning and species interactions into consideration. The general form of c_i is expressed as follows

$$c_i = e_i - \sum_{k=1}^{m} x_{ki} s_k \qquad (5)$$

where e_i is the maximum carrying capacity of the niche where species i locates, and x_{ki} represents the strength of the interaction that species k imposes on species i. In its simplest case, we applied the logistic growth model, which means that we only considered the competition among species. Similar to the way in which we characterize plasmid incompatibility, we distributed the m species into r niches W_1, W_2,..., W_r ($r \leq m$). The maximum carrying capacity of these niches are y_1, y_2,..., y_r, respectively. Let V_i represent the map from the species index i to its niche index. Therefore, $e_i = y_{V_i}$. Then, the formulation of C_i that we used in our analysis was obtained as

$$c_i = y_{V_i} - \sum_{\{k:\, V_k = V_i\}} s_k. \qquad (6)$$

The ODEs, and the formulations of α, β and c, constitute the body of our framework.

2.1.3 Numerical simulations to test the persistence potential

We used the pipeline shown in the flowchart below to conduct the numerical simulations of HGT dynamics in microbial communities. In particular, we configured each simulation by using randomized parameters, as listed below (each follows a uniform distribution within the specified range:

number of species m: 5-100;

number of plasmids n: 1-50;

number of niches: from 1 to the total number of species;

carrying capacity for each niche e: from 0 to 1, the carrying capacity of the niches were then normalized so that the total carrying capacities of all the niches equaled 1;

maximum growth rate μ: 0.4-0.8 per hour;

dilution rate D: 0.001-0.004 per hour;

plasmid segregation loss rate κ: 0-0.002 per hour;

plasmid burden λ: 0-0.2;

plasmid transfer rate η: 0-0.02 per hour.

To simulate the system dynamics, we first randomized the number of species and plasmids in each community. We then generated a random number of the niches (denoted as k) and allocated each species into one of the niches. When allocating the species, each niche was given a random number γ_i following a uniform distribution between 0 and 1. Then for each species, we generated a random number ϵ following a uniform distribution between 0 and 1. If $\frac{\sum_{i=1}^{j-1}\gamma_i}{\sum_{i=1}^{k}\gamma_i} < \epsilon \leq \frac{\sum_{i=1}^{j}\gamma_i}{\sum_{i=1}^{k}\gamma_i}$, the species will be allocated into the j-th niche. The niches without any species were removed and each remaining niche was assigned a random value of the maximum carrying capacity. Then the carrying capacities of the niches were normalized so that the total carrying capacity of all the niches equaled 1. Next, the maximum rates of the species and the dilution rate were randomized. The dilution rate was same for each species or plasmid in the same community. For plasmid dynamics, the transfer rates of each plasmid were randomized in two steps: (1) the transfer rate between a specific pair of species might be zero or no-zero, by a random chance that uniformly distributes from 0 to 1; (2) if the transfer rate is non-zero, the value of η is also randomized. Finally, the plasmid loss rates, the growth effects, as well as the initial densities of each population were randomized. All the parameters follow a uniform distribution within the specified ranges described above.

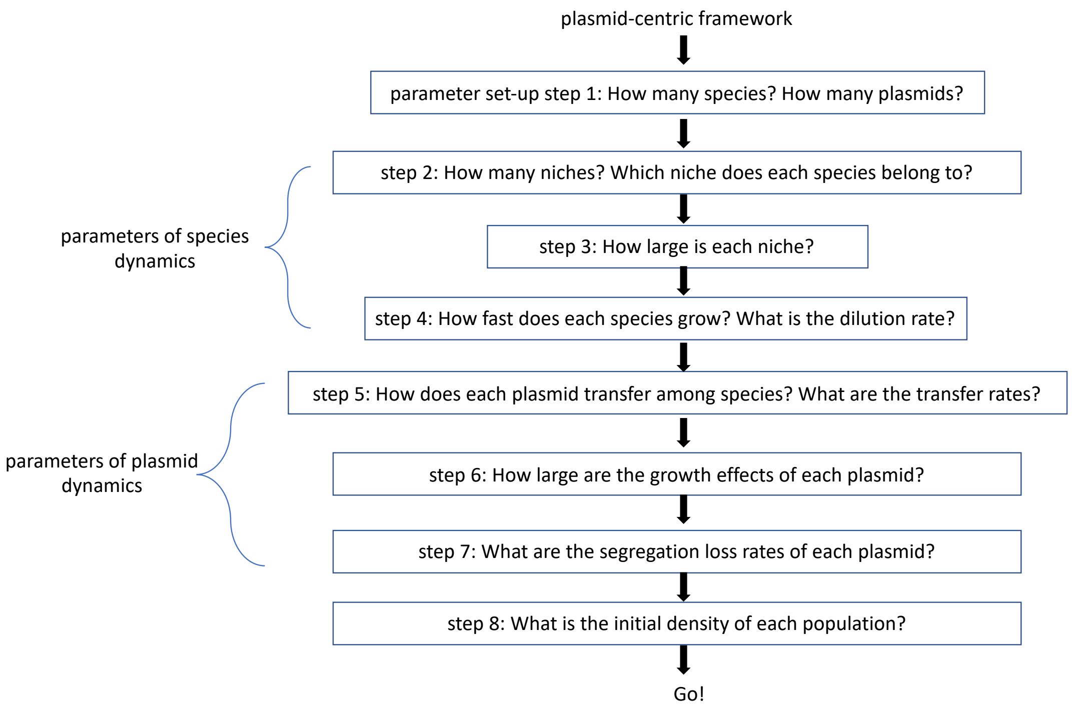

With all these parameters, we then were able to simulate the temporal dynamics of the microbial communities. The timespan of our simulations was 0-30,000 hours, which was sufficiently long for the system to reach the steady state. We then calculated the steady-state abundance of each plasmid and the corresponding persistence potential. The results were shown in the Fig. 3c of the main text. Here, the assumption of steady state is not necessary for the prediction power of the persistence potential. The ω values can have similar predictive power for the plasmid abundance well before the system has reached equilibrium state. One example was shown in Supplementary Fig. 3.

2.2 Analysis on the computation complexities of subpopulation-centric framework (SCF) and plasmid-centric framework (PCF)

Let's consider a microbial community of m species and n mobilizable plasmids. In SCF, the population can be divided into $m \cdot 2^n$ subpopulations. Each subpopulation represents a unique combination of species and plasmids. Thus, SCF requires $m \cdot 2^n$ ODEs. However, PCF contains only $m(n+1)$ ODEs, m of which describe population dynamics, and mn of which describe plasmid dynamics.

The computation complexities of these two frameworks can also be quantified by the number of parameters. In SCF, each subpopulation has its unique growth rate. Thus, $m \cdot 2^n$ growth rates are required. For each of the n plasmids, there are $m \cdot 2^{n-1}$ subpopulations that carry it and another $m \cdot 2^{n-1}$ subpopulations that do not carry it. The total number of transfer rates can then be obtained as $n(m \cdot 2^{n-1})^2 = nm^2 \cdot 2^{2n-2}$. Every subpopulation that carries the specific plasmid has a unique rate of losing it. Therefore, the number of plasmid loss rates in SCF is $mn \cdot 2^{n-1}$.

In PCF, because we only considered the growth rates of the plasmid-free cells as well as the fitness cost of each individual plasmid, $m(n+1)$ growth rate constants are needed. For horizontal transfer, we only considered the transfer rates of a specific plasmid across different species, so the total number of transfer rates was also greatly reduced to nm^2. In a similar way, for each plasmid, we only considered its loss in each species instead of in each subpopulation, so the number of loss rates was reduced to mn. The results of this computational complexity analysis are summarized in Supplementary Table 2.

Supplementary Table 2 Analysis of the computation complexities of SCF and PCF

		SCF	PCF
Number of ODEs		$m \cdot 2^n$	$m(n+1)$
Number of parameters	Growth rates	$m \cdot 2^n$	$m(n+1)$
	Segregation loss rates	$mn \cdot 2^{n-1}$	mn
	Dilution rates	1	1
	Conjugation efficiencies	$nm^2 \cdot 2^{2n-2}$.	nm^2

2.3 Discrepancy between SCF and PCF

For microbial communities with multiple species and one plasmid, we compared the performances of the two frameworks using numerical simulations. We assigned the same sets of randomized parameters to both models. The relative abundances of plasmids, quantified as the fractions of plasmid-carrying cells at steady states, from the two models were aligned with each other (Supplementary Fig. 2a). From 1000 repeated numerical tests, no qualitative discrepancy

was detected. The quantitative discrepancy shows dependence on fitness cost. Lower average growth burden or benefit gives rise to better match.

For communities with multiple coexisting plasmids, SCF requires more parameters to predict the plasmid dynamics (Supplementary Table 2). Here we focus on a community with one species and two plasmids (denoted A and B, respectively). Let λ_A and λ_B represent the individual fitness costs when the plasmids exist separately. In PCF, the combined fitness cost of A and B when they coexist in the same host cell can be derived from λ_A and λ_B. However, in SCF, one additional parameter λ_{AB} is required to characterize the combined effect. We calculated the outputs under different values of λ_A and λ_B as well as λ_{AB} (Supplementary Fig. 2b). The results suggest that the two models match well over a wide range of fitness costs.

2.4 Development of plasmid persistence potential

We started with a microbial community of m species and one plasmid. Assuming that the community is homogeneous, i.e. all the species carry the same kinetic parameters, the dynamics of this community can be described by $2m$ ODEs:

$$\frac{ds_i}{dt} = \frac{s_i}{s_i + \lambda p_i} \mu s_i \left(\frac{1}{m} - s_i\right) - D s_i \quad (7)$$

$$\frac{dp_i}{dt} = \frac{\mu}{1+\lambda} p_i \left(\frac{1}{m} - s_i\right) + m\eta(s_i - p_i)p_i - (D + \kappa)p_i \quad (8)$$

$$(i = 1,2,\dots,m).$$

We then assumed that all s_i or p_i start with the same initial densities ($s_1^0 = s_2^0 = \cdots = s_m^0$ and $p_1^0 = p_2^0 = \cdots = p_m^0$). Therefore, at any time points, $s_1 = s_2 = \cdots = s_m$ and $p_1 = p_2 = \cdots = p_m$. Thus, this system can be simplified as two ODEs:

$$\frac{dx}{dt} = \frac{x}{x + \lambda y} \mu_e x(1 - x) - Dx \quad (9)$$

$$\frac{dy}{dt} = \frac{\mu_e}{1+\lambda} y(1 - x) + \eta(x - y)y - (D + \kappa)y \quad (10)$$

where $x = ms_i$, $y = mp_i$ and $\mu_e = \frac{\mu}{m}$. At steady state, with $x \neq 0$ and $y \neq 0$, Eq. 9 can be transformed to

$$y = \frac{-\mu_e x^2 + (\mu_e - D)x}{D\lambda} \qquad (11)$$

and Eq. 10 can be transformed to

$$y = \left(1 - \frac{\mu_e}{\alpha\eta}\right)x + \left(\frac{\mu_e}{\alpha\eta} - \frac{D+\kappa}{\eta}\right). \qquad (12)$$

To derive the criterion of plasmid persistence in this simplified case, we then sought the conditions under which Eq. 11 and Eq. 12 give rise to solutions with positive and feasible values of x and y ($0 < \mathrm{x} \leq 1, 0 < \mathrm{y} \leq \mathrm{x}$). Mathematically, this is a quadratic problem, and the criterion can be analytically derived out as follows

$$\eta > \frac{\mu}{\mu - mD} \cdot \frac{\alpha(D+\kappa) - D}{\alpha}. \qquad (13)$$

We defined the plasmid persistence potential as

$$\omega = \frac{\eta}{\frac{\mu}{\mu - mD} \cdot \frac{\alpha(D+\kappa) - D}{\alpha}}. \qquad (14)$$

Therefore, this criterion can be expressed as $\omega > 1$.

Next, we sought to generalize the criterion to heterogeneous communities. We reformulated ω in the following manner:

$$\omega = \frac{\bar{\eta}}{\frac{\bar{\mu}}{\bar{\mu} - \sigma D}\left(D + \bar{\kappa} - \frac{D}{1+\bar{\lambda}}\right)} \qquad (15)$$

where $\bar{\mu} = \sum_{i=1}^{m} \frac{s_i}{s_T}\mu_i$, $\bar{\kappa} = \sum_{i=1}^{m} \frac{s_i}{s_T}\kappa_i$, $\bar{\lambda} = \sum_{i=1}^{m} \frac{s_i}{s_T}\lambda_i$, and $\bar{\eta} = \sum_{i=1}^{m}\sum_{j=1}^{m} \frac{s_i}{s_T}\frac{s_j}{s_T}\eta_{ij}$. s_T is the total abundance of all the populations: $s_T = \sum_{i=1}^{m} s_i$. σ is the Shannon effective number of species: $\sigma = e^{-\sum_{i=1}^{m}\frac{s_i}{s_T}ln\frac{s_i}{s_T}}$

2.5 The application of the framework to lytic bacteriophage and transposons

2.5.1 Lytic bacteriophages

During infection, lytic bacteriophages replicate in the host cells. After a short latent time, the infected host cells lyse, releasing the resident phages[2,3]. Because the host cannot grow in the lytic cycle, our framework requires modifications to accommodate lytic phage dynamics. The new ODEs are shown below:

$$\frac{ds}{dt} = \mu s(1 - s) - \eta sp - Ds \qquad (16)$$

$$\frac{dp}{dt} = \beta\eta sp - Dp - \kappa p. \qquad (17)$$

s and p stand for the densities of cells and phages, respectively. μ is the growth rate constant of cells. η is the rate constant at which phages encounter and irreversibly infect the cells. One infected cell releases β phages after lysing. D and κ represent the dilution and phage loss rates, respectively.

This model represents a simplified version of the classic one[2,3]. The condition for phage persistence ($q > 0$) can be derived as

$$\beta\eta > \frac{\mu}{\mu - D}(D + \kappa). \qquad (18)$$

Therefore, we defined the persistence potential in this case as

$$\omega = \frac{\beta\eta}{\frac{\mu}{\mu - D}(D + \kappa)}. \qquad (19)$$

For heterogeneous communities, the formulation of ω can be generalized as described in Section 2.4.

2.5.2 Transposons

Transposons are the genetic elements that locate on chromosomes or plasmids. The dynamical processes of transposons are more complex than those of plasmids. The transfer of transposons is mediated by plasmid conjugation, and the intercellular transfer of the transposon includes two steps: (1) the transposition of the transposon from chromosome to plasmid and (2) the conjugation of the plasmid across different cells[4].

For a population in which all cells carry the conjugative plasmid, let s_i ($i = 1,2, \dots, m$) represent the abundance of the species i, and p_{ij} ($i = 1,2, \dots, m$ and $j = 1,2, \dots, n$) represent the abundance of transposon j-carrying cells in the species i. The overall transfer rate of the transposon is represented by η. λ_{ij} stands for the fitness cost of the jth transposon on the ith species. The transposon can be lost through plasmid segregation loss and excision from chromosome or plasmid[5]. The rate constant of transposon loss is represented by κ. The transposon dynamics can be described by the same ODEs described in Section 2.1.2.

Section 3: Analysis of literature data

Here, we summarized the data from 9 previous studies in the literature. The microbial communities analyzed in these studies covered one, two, or three populations, and up to three plasmids. The data sources of these data are shown in Supplementary Table 3.

Supplementary Table 3 Summary of the referred papers

References	Species number	Plasmid number
Hall JP *et al*, 2016[6].	1,2	1
Lopatkin AJ *et al*, 2017[1].	1,2,3	1,2,3
Loftie-Eaton W *et al*, 2017[7].	1	1
Hall JP *et al*, 2017[8].	2	1
Harrison E *et al*, 2015[9].	1	1
Kottara A *et al*, 2017[10].	1	1
Dahlberg C *et al*, 2003[11].	1	1
Fischer EA *et al*, 2014[12] .	1,2	1
Porse A *et al*, 2016[13].	1	1

3.1 Source-sink plasmid transfer dynamics maintain gene mobility in soil bacterial communities

In this study, Hall JP *et al*. cultured two strains *Pseudomonas fluorescens* SBM25 and *Pseudomonas putica* KT2440 either individually or together. In each experiment, the populations started with 50% frequency of a mercury resistance plasmid, pQBR57. The samples were transferred into fresh media either with or without mercuric chloride selections. The dynamics of the bacteria populations were tracked over 65 transfers.

We calculated the ω values and the corresponding relative abundance of plasmid pQBR57 in each of the experiments. Because the parameter values under mercuric chloride treatment were lacking, we only focused on the experimental data without mercuric selections. Additionally, we only considered the data after transfer 29 when the systems were close to steady states. With these two constraints, the original data we reused in this paper are summarized in Supplementary Table 4. The parameter values are summarized in Supplementary Table 5. The λ factors in our model can be calculated from the α values shown in Supplementary Table 5 by $\lambda_1 = \frac{1}{\alpha_1} - 1$, $\lambda_2 = \frac{1}{\alpha_2} - 1$. The conjugation rates were measured using the end-point method, with units of $cell^{-1}hr^{-1}$, and was dependent on the cell density. In our model, we used the cell density-independent conjugation rate constant η, by normalizing the measured values with respect to the maximum carrying capacities N_m. For the experiments with single species, N_m values are shown in Supplementary Table 5. For coculture experiments, we estimated N_m as the mean value of total population size. In this work, cell death instead of dilution was considered. Since both death rate d and dilution rate D occupy the same position in our equations, the persistence potential can be calculated as $\omega = \frac{\bar{\eta}}{\frac{\bar{\mu}}{\bar{\mu}-\sigma d}\left(d+\bar{\kappa}-\frac{d}{1+\bar{\lambda}}\right)}$. The pQBR57 relative abundance was calculated as the ratio of plasmid-carrying cells to the total population size. The ω values and the corresponding abundances are summarized in Supplementary Table 6.

Supplementary Table 4. The original data of the relative abundance of plasmid pQBR57 from 'Source-sink plasmid transfer dynamics maintain gene mobility in soil bacterial communities.'

		single species				coculture			
		Pseudomonas fluorescens		*Pseudomonas putica*		*Pseudomonas fluorescens*		*Pseudomonas putica*	
		Cell density	Plasmid density	Cell density	Plasmid density	Cell density	Plasmid density	Cell density	Plasmid density
Transfer 29	A	7.1×10^8	6.9×10^8	2.4×10^8	0	1×10^9	0	1.2×10^8	0
	B	5×10^8	5×10^8	1.6×10^8	0	5×10^8	0	5×10^7	0
	C	6.1×10^8	4.2×10^8	1.2×10^8	0	6×10^8	3.7×10^8	1.5×10^8	0
	D	9.1×10^8	2.8×10^7	5.4×10^7	0	8.8×10^8	0	9.5×10^7	0
	E	5.2×10^8	1.6×10^7	1.1×10^8	0	5.5×10^8	5.4×10^8	5.3×10^7	1.7×10^6
	F	4.9×10^8	3.2×10^8	1.2×10^8	0	6.4×10^8	2×10^7	5×10^7	4.7×10^6
Transfer 35	A	6×10^8	6×10^8	9×10^7	0	3.6×10^8	0	1.4×10^7	0
	B	4.4×10^8	4×10^8	8.7×10^7	0	4.8×10^8	4.5×10^7	4.2×10^7	0

	C	6.2×10^8	4.5×10^8	1.3×10^8	0	3.9×10^8	3.9×10^8	3.1×10^7	0
	D	3.6×10^8	3×10^8	9.8×10^7	3.1×10^6	4.5×10^8	0	7×10^7	0
	E	5×10^8	5×10^8	6.8×10^7	0	3.2×10^8	3.2×10^8	7×10^7	1.8×10^7
	F	5.1×10^8	4.1×10^8	8.6×10^7	0	3.4×10^8	1.7×10^8	2.5×10^7	7.9×10^5
Transfer 41	A	4.9×10^8	4.9×10^8	5.6×10^7	0	4.8×10^8	0	5.6×10^7	0
	B	4.8×10^8	4.7×10^8	8.8×10^7	0	2.3×10^8	2×10^8	2.5×10^7	3.2×10^6
	C	3.7×10^8	3.4×10^8	1.3×10^8	0	3.4×10^8	3.3×10^8	6.4×10^7	2×10^6
	D	4.6×10^8	4.6×10^8	1.4×10^8	0	4.1×10^8	3×10^8	4.5×10^7	2.8×10^6
	E	4.6×10^8	4.6×10^8	9.2×10^7	0	5×10^8	4.8×10^8	7×10^7	3.5×10^7
	F	3.9×10^8	3.8×10^8	7.4×10^7	0	4.8×10^8	4.5×10^8	3.9×10^7	3.7×10^6
Transfer 47	A	5.74×10^8	5.74×10^8	1.4×10^8	0	4.9×10^8	0	5.3×10^7	0
	B	6.8×10^8	6.6×10^8	1.4×10^8	0	6.7×10^8	4×10^8	7.3×10^7	2.3×10^6
	C	4.9×10^8	4.9×10^8	1.2×10^8	0	4.4×10^8	4.4×10^8	3.9×10^7	0
	D	5.04×10^8	5.04×10^8	1.4×10^7	0	5.3×10^8	3.6×10^8	4.2×10^7	1.3×10^6
	E	6×10^8	6×10^8	1.1×10^8	0	5.9×10^8	5.9×10^8	9.5×10^7	3×10^7
	F	6.2×10^8	6×10^8	1.2×10^7	0	6.2×10^8	4.7×10^8	2.8×10^7	1.8×10^6
Transfer 53	A	6.2×10^8	5.2×10^8	1.1×10^8	3.4×10^6	5.3×10^8	0	6.9×10^7	0
	B	4.8×10^8	4.3×10^8	1.1×10^8	0	4.1×10^8	3.8×10^8	6.6×10^7	0
	C	4.5×10^8	4.2×10^8	1.2×10^8	0	4.6×10^8	2.9×10^8	1.5×10^8	9.1×10^6
	D	5×10^8	5×10^8	8.6×10^7	0	3.4×10^8	2.1×10^8	4×10^7	2.5×10^6
	E	4.8×10^8	4.8×10^8	1×10^8	0	3.6×10^8	3.6×10^8	6.9×10^7	2.2×10^7
	F	5×10^8	4.7×10^8	9.4×10^7	0	4.4×10^8	4×10^8	3.6×10^7	2.3×10^6
Transfer 59	A	4.7×10^8	4.7×10^8	5.2×10^7	0	5.8×10^8	0	5.9×10^7	0
	B	4.7×10^8	3.9×10^8	9.1×10^7	0	4.9×10^8	4.8×10^8	3.6×10^7	2.3×10^6
	C	5.1×10^8	5×10^8	1.1×10^8	0	4.2×10^8	2.6×10^8	6.3×10^7	0
	D	3.7×10^8	3.7×10^8	3.9×10^7	0	4.3×10^8	3.5×10^8	5.6×10^7	1.8×10^6
	E	4×10^8	4×10^8	8.1×10^7	0	3.3×10^8	3.3×10^8	3.6×10^7	0
	F	3.9×10^8	3.7×10^8	1×10^8	0	4×10^8	2.7×10^8	8.3×10^7	0
Transfer 65	A	3.2×10^8	3.2×10^8	1.3×10^8	0	2.9×10^8	0	1.7×10^8	0
	B	7.9×10^8	7.2×10^8	1.6×10^8	0	2.6×10^8	2×10^8	1.1×10^8	0
	C	5.4×10^8	4.9×10^8	1.3×10^8	0	5.4×10^8	1.9×10^8	1.1×10^8	3.4×10^6
	D	3.6×10^8	3.1×10^8	1.5×10^8	0	3.4×10^8	1.4×10^8	9.6×10^7	0
	E	6.5×10^8	6.3×10^8	1.2×10^8	0	4.4×10^8	4.4×10^8	1.8×10^8	2.3×10^7
	F	6.3×10^8	3.1×10^8	1.2×10^8	0	3.6×10^8	9×10^7	8.6×10^7	0

* The plasmid abundance data, which were visualized in Fig. 1a and c of this referred paper, were obtained from DRYAD (https://datadryad.org/bitstream/handle/10255/dryad.119190/Figure1Data.csv?sequence=1).

Supplementary Table 5 The parameter values used in 'Source-sink plasmid transfer dynamics maintain gene mobility in soil bacterial communities'

Name	Variable measured	Estimate
μ_1	*Pseudomonas fluorescens* growth rate	$0.091\ h^{-1}$
μ_2	*Pseudomonas putica* growth rate	$0.186\ h^{-1}$
α_1	Effect of plasmid carriage on *Pseudomonas fluorescens* growth rate	0.73
α_2	Effect of plasmid carriage on *Pseudomonas putica* growth rate	0.85
η_{11}	Intraspecific conjugation rate in *Pseudomonas fluorescens*	$10^{-11}\ cell^{-1} \cdot h^{-1}$
η_{12}	Interspecific conjugation rate from *Pseudomonas fluorescens* to *Pseudomonas putica*	$10^{-14}\ cell^{-1} \cdot h^{-1}$
η_{21}	Interspecific conjugation rate from *Pseudomonas putica* to *Pseudomonas fluorescens*	$10^{-11.5}\ cell^{-1} \cdot h^{-1}$
η_{22}	Intraspecific conjugation rate in *Pseudomonas putica*	$10^{-14}\ cell^{-1} \cdot h^{-1}$
κ	*Segregation rate*	$1 \times 10^{-4}\ h^{-1}$
N_{m1}	Carrying capacity of *Pseudomonas fluorescens*	6.01×10^{8}
N_{m2}	Carrying capacity of *Pseudomonas putica*	1.1×10^{8}
d	Death rate	$0.009\ h^{-1}$

* The parameter values were obtained from Supplementary Table 1 of this referred paper. We changed the letters of each parameter to be consistent with our model notations.

Supplementary Table 6 Plasmid pQBR57 persistence potential and the corresponding relative abundance in 'Source-sink plasmid transfer dynamics maintain gene mobility in soil bacterial communities'

<table>
<tr><th colspan="3"></th><th>Plasmid persistence potential</th><th>Plasmid relative abundance</th></tr>
<tr><td rowspan="3">Transfer 29</td><td rowspan="2">Single species</td><td>Pseudomonas fluorescens</td><td>2.14</td><td>0.56 ± 0.43</td></tr>
<tr><td>Pseudomonas putica</td><td>7.22×10^{-4}</td><td>0</td></tr>
<tr><td colspan="2">Coculture</td><td>1.39 ± 0.08</td><td>0.24 ± 0.37</td></tr>
<tr><td rowspan="3">Transfer 35</td><td rowspan="2">Single species</td><td>Pseudomonas fluorescens</td><td>2.14</td><td>0.88 ± 0.11</td></tr>
<tr><td>Pseudomonas putica</td><td>7.22×10^{-4}</td><td>0.005 ± 0.013</td></tr>
<tr><td colspan="2">Coculture</td><td>1.41 ± 0.09</td><td>0.39 ± 0.43</td></tr>
<tr><td rowspan="3">Transfer 41</td><td rowspan="2">Single species</td><td>Pseudomonas fluorescens</td><td>2.14</td><td>0.98 ± 0.03</td></tr>
<tr><td>Pseudomonas putica</td><td>7.22×10^{-4}</td><td>0</td></tr>
<tr><td colspan="2">Coculture</td><td>1.39 ± 0.05</td><td>0.68 ± 0.34</td></tr>
</table>

Transfer 47	Single species	*Pseudomonas fluorescens*	2.14	0.99 ± 0.02
		Pseudomonas putica	7.22×10^{-4}	0
	Coculture		1.42 ± 0.06	0.62 ± 0.34
Transfer 53	Single species	*Pseudomonas fluorescens*	2.14	0.96 ± 0.04
		Pseudomonas putica	7.22×10^{-4}	0.005 ± 0.013
	Coculture		1.36 ± 0.10	0.59 ± 0.33
Transfer 59	Single species	*Pseudomonas fluorescens*	2.14	0.96 ± 0.06
		Pseudomonas putica	7.22×10^{-4}	0
	Coculture		1.38 ± 0.06	0.61 ± 0.34
Transfer 65	Single species	*Pseudomonas fluorescens*	2.14	0.86 ± 0.19
		Pseudomonas putica	7.22×10^{-4}	0
	Coculture		1.13 ± 0.13	0.35 ± 0.26

3.2 Persistence and reversal of plasmid-mediated antibiotic resistance

Lopatkin *et al*. studied the conjugation-assisted persistence of different plasmids. Here we focused on three groups of experiments presented in their paper.

(1) They introduced a mobilizable plasmid (denoted plasmid K) into the *E.coli* strain MG1655 (denoted strain B) equipped with a helper plasmid F_{HR}. F_{HR} is not self-transmissible but encodes the conjugation machinery and helps the transfer of plasmid K (kanamycin-resistant). They then quantified the long-term dynamics of plasmid K abundance. Different antibiotic concentrations were applied to change the fitness of plasmid K. Another mobilizable plasmid denoted C (Chloramphenicol-resistant) that carries mCherry marker was also tested in strain B. They also quantified the dynamics of plasmids 168, 193, R388, 41, RP4, PCU1, and R6K in an *E.coli* MG1655 strain with chromosomally integrated dTomato (denoted as strain R).

(2) They used linoleic acid to inhibit conjugation and phenothiazine to enhance the plasmid segregation error.

(3) They tested plasmid persistence in complex communities: (a) strain B mixed with strain R, carrying plasmid K; (b) strain B, carrying plasmids K and C; (c) strain B, strain R, and strain Y (*E.coli* MG1655 with YFP), carrying plasmids R6K, RP4, and R388. In experiments (a) and (b), different concentrations of kanamycin or chloramphenicol were added to change the fitness cost of the plasmids.

The kinetic parameters of these experiments are summarized in Supplementary Table 7. The α values represent the magnitude of plasmid burdens. The λ factors in our model can be calculated from the α values through $\lambda = \alpha - 1$. To transform the measured values of conjugation efficiency η to the values used in their mathematical model, Lopatkin *et al.* normalized η respect to the maximum carrying capacity $N_m = 1 \times 10^9\ cell \cdot mL^{-1}$. However, in these experiments, η was measured using cells in stationary phase, so they accounted for the physiological influence on η by multiplying the measured conjugation efficiency by a factor of 2.5×10^3. In this way, we calculated the plasmid persistence potential in these experiments, as shown in Supplementary Table 8.

Supplementary Table 7 Parameter values used in 'Persistence and reversal of plasmid-mediated antibiotic resistance'

Name	Variable measured	Estimate
μ_B^K	Growth rate of B^K population	$0.3\ h^{-1}$
μ_R^K	Growth rate of R^K population	$0.29\ h^{-1}$
μ_B^C	Growth rate of B^C population	$0.28\ h^{-1}$
μ_R^{41}	Growth rate of R^{41} population	$0.14\ h^{-1}$
μ_R^{168}	Growth rate of R^{168} population	$0.19\ h^{-1}$
μ_R^{193}	Growth rate of R^{193} population	$0.21\ h^{-1}$
μ_R^{RP4}	Growth rate of R^{RP4} population	$0.20\ h^{-1}$
μ_R^{R6K}	Growth rate of R^{R6K} population	$0.25\ h^{-1}$
μ_R^{PCU1}	Growth rate of R^{PCU1} population	$0.11\ h^{-1}$
μ_R^{R388}	Growth rate of R^{PCU1} population	$0.28\ h^{-1}$
α_B^K	Growth burden of plasmid K on strain B	1.02
$\alpha_B^{K'}$	Growth burden of plasmid K on strain B with 0.5 $\mu g/ml$ Kanamycin treatment	0.97
$\alpha_B^{K''}$	Growth burden of plasmid K on strain B with 2 $\mu g/ml$ Kanamycin treatment	0.42
$\alpha_B^{K'''}$	Growth burden of plasmid K on strain B with 0.5 $\mu g/ml$ Chloramphenicol treatment	1.035
$\alpha_B^{K''''}$	Growth burden of plasmid K on strain B with 2 $\mu g/ml$ Chloramphenicol treatment	1.00
α_R^K	Growth burden of plasmid K on strain R	1.07
$\alpha_R^{K'}$	Growth burden of plasmid K on strain R with 0.5 $\mu g/ml$ Kanamycin treatment	0.99
$\alpha_R^{K''}$	Growth burden of plasmid K on strain R with 2 $\mu g/ml$ Kanamycin treatment	0.86
α_B^C	Growth burden of plasmid C on strain B	1.21
$\alpha_B^{C'}$	Growth burden of plasmid C on strain B with 0.5 $\mu g/ml$ Chloramphenicol treatment	0.89
$\alpha_B^{C''}$	Growth burden of plasmid C on strain B with 2 $\mu g/ml$ Chloramphenicol treatment	0.33

$\alpha_B^{C'''}$	Growth burden of plasmid C on strain B with 0.5 $\mu g/ml$ Kanamycin treatment	1.5
$\alpha_B^{C''''}$	Growth burden of plasmid C on strain B with 2 $\mu g/ml$ Kanamycin treatment	1.00
α_R^{C}	Growth burden of plasmid C on strain R	1.22
α_R^{41}	Growth burden of plasmid 41 on strain R	1.37
α_R^{168}	Growth burden of plasmid 168 on strain R	0.97
α_R^{193}	Growth burden of plasmid 193 on strain R	0.90
α_R^{RP4}	Growth burden of plasmid RP4 on strain R	0.90
α_B^{RP4}	Growth burden of plasmid RP4 on strain B	0.89
α_Y^{RP4}	Growth burden of plasmid RP4 on strain Y	0.90
α_R^{R6K}	Growth burden of plasmid R6K on strain R	0.75
α_B^{R6K}	Growth burden of plasmid R6K on strain B	0.87
α_Y^{R6K}	Growth burden of plasmid R6K on strain Y	0.92
α_R^{PCU1}	Growth burden of plasmid PCU1 on strain R	2.80
α_R^{R388}	Growth burden of plasmid R388 on strain R	0.66
α_B^{R388}	Growth burden of plasmid R388 on strain B	0.84
α_Y^{R388}	Growth burden of plasmid R388 on strain Y	0.86
η_K	Conjugation efficiency of plasmid K without inhibition	$2.5 \times 10^{-15}\ cell^{-1}h^{-1}mL$
η_C	Conjugation efficiency of plasmid C without inhibition	$1.09 \times 10^{-14}\ cell^{-1}h^{-1}mL$
η_{41}	Conjugation efficiency of plasmid 41 without inhibition	$1.80 \times 10^{-14}\ cell^{-1}h^{-1}mL$
η_{168}	Conjugation efficiency of plasmid 168 without inhibition	$5.94 \times 10^{-15}\ cell^{-1}h^{-1}mL$
η_{193}	Conjugation efficiency of plasmid 193 without inhibition	$2.38 \times 10^{-15}\ cell^{-1}h^{-1}mL$
η_{RP4}	Conjugation efficiency of plasmid RP4 without inhibition	$1.76 \times 10^{-12}\ cell^{-1}h^{-1}mL$
η_{R6K}	Conjugation efficiency of plasmid R6K without inhibition	$9.19 \times 10^{-14}\ cell^{-1}h^{-1}mL$
η_{PCU1}	Conjugation efficiency of plasmid PCU1 without inhibition	$1.45 \times 10^{-13}\ cell^{-1}h^{-1}mL$
η_{R388}	Conjugation efficiency of plasmid R388 without inhibition	$1.94 \times 10^{-12}\ cell^{-1}h^{-1}mL$
D	Dilution rate	$0.05\ h^{-1}$
κ	Segregation loss rate without phenothiazine	$5.2 \times 10^{-4}\ h^{-1}$
σ_K	Fold-decrease of conjugation efficiency of plasmid K with inhibition	2.96
σ_C	Fold-decrease of conjugation efficiency of plasmid C with inhibition	1.54
σ_{41}	Fold-decrease of conjugation efficiency of plasmid 41 with inhibition	12.43
σ_{168}	Fold-decrease of conjugation efficiency of plasmid 168 with inhibition	53.42
σ_{193}	Fold-decrease of conjugation efficiency of plasmid 193 with inhibition	3.31
σ_{RP4}	Fold-decrease of conjugation efficiency of plasmid RP4 with inhibition	13.06
σ_{R6K}	Fold-decrease of conjugation efficiency of plasmid R6K with inhibition	2.46
σ_{PCU1}	Fold-decrease of conjugation efficiency of plasmid PCU1 with inhibition	13.09
σ_{R388}	Fold-decrease of conjugation efficiency of plasmid PCU1 with inhibition	2.49
κ_{Ph}	Segregation loss rate with phenothiazine	$2.1 \times 10^{-3}\ h^{-1}$

*The parameter values were obtained from Supplementary Tables 2, 3, Supplementary Fig. 3 and 6 of this referred paper. We changed the letters of each parameters to be consistent with our model notations.

Supplementary Table 8 Persistence potential and the corresponding relative abundance of plasmid RP4 in 'Persistence and reversal of plasmid-mediated antibiotic resistance'

	Plasmid persistence potential ω	Plasmid relative abundance	Description
Simple communities without linoleic acid and phenothiazine	1.38	1 ± 0.00	Plasmid K in strain R
	2.53	0.96 ± 0.07	Plasmid C in strain B (selective plating)
	2.37	1.02 ± 0.06	Plasmid 41 in strain R
	-10.54	0.91 ± 0.07	Plasmid 168 in strain R
	-0.87	1.01 ± 0.06	Plasmid 193 in strain R
	-625	0.97 ± 0.21	Plasmid RP4 in strain R
	-10.44	0.91 ± 0.04	Plasmid R6K in strain R
	-140.85	0.90 ± 0.04	Plasmid R388 in strain R
Simple communities with linoleic acid and phenothiazine	0.33	0 ± 0.00	Plasmid K in strain R
	1.40	0.28 ± 0.03	Plasmid C in strain B
	0.17	0 ± 0.00	Plasmid 41 in strain R
	0.38	0.02 ± 0.00	Plasmid 168 in strain R
	-0.38	0.43 ± 0.03	Plasmid 193 in strain R
	-69.93	0.66 ± 0.12	Plasmid RP4 in strain R
	-4.69	0.60 ± 0.08	Plasmid R6K in strain R
	0.68	0 ± 0.00	Plasmid PCU1 in strain R
	-59.88	0.81 ± 0.04	Plasmid R388 in strain R
Simple communities with antibiotic treatment	3.48	1.00 ± 0.00	Plasmid K in strain B with 0 $\mu g/ml$ Kanamycin
	-5.04	1.00 ± 0.01	Plasmid K in strain B with 0.5 $\mu g/ml$ Kanamycin
	-0.06	1.00 ± 0.00	Plasmid K in strain B with 2 $\mu g/ml$ Kanamycin
	2.53	0.69 ± 0.16	Plasmid C in strain B with 0 $\mu g/ml$ Chloramphenicol (flow cytometry)
	-3.85	0.90 ± 0.04	Plasmid C in strain B with 0.5 $\mu g/ml$ Chloramphenicol
	-0.12	0.98 ± 0.02	Plasmid C in strain B with 2 $\mu g/ml$ Chloramphenicol
Complex communities	1.96	0.98 ± 0.04	Plasmid K in community B^K+R^K with 0 $\mu g/ml$ Kanamycin
	-10.33	0.99 ± 0.05	Plasmid K in community B^K+R^K with 0.5 $\mu g/ml$ Kanamycin
	-0.17	0.97 ± 0.02	Plasmid K in community B^K+R^K with 2 $\mu g/ml$ Kanamycin
	3.48	0.95 ± 0.06	Plasmid K in community B^K+B^C+B^{CK} with 0 $\mu g/ml$ Kanamycin

	-5.04	0.97 ± 0.06	Plasmid K in community B^K+B^C+B^{CK} with0.5 $\mu g/ml$ Kanamycin
	-0.06	0.97 ± 0.02	Plasmid K in community B^K+B^C+B^{CK} with 2 $\mu g/ml$ Kanamycin
	2.37	0.91 ± 0.15	Plasmid K in community B^K+B^C+B^{CK} with 0.5 $\mu g/ml$ Chloramphenicol
	10.02	0.82 ± 0.07	Plasmid K in community B^K+B^C+B^{CK} with2 $\mu g/ml$ Chloramphenicol
	2.53	0.50 ± 0.23	Plasmid C in community B^K+B^C+B^{CK} with 0 $\mu g/ml$ Kanamycin
	1.40	0.24 ± 0.28	Plasmid C in community B^K+B^C+B^{CK} with0.5 $\mu g/ml$ Kanamycin
	-3.85	0.83 ± 0.11	Plasmid C in community B^K+B^C+B^{CK} with 0.5 $\mu g/ml$ Chloramphenicol
	-0.12	0.84 ± 0.05	Plasmid C in community B^K+B^C+B^{CK} with 2 $\mu g/ml$ Chloramphenicol
	-20.49	0.22 ± 0.00	Plasmid R6K in 3-species (B, R, Y) 3-plasmid (R6K+R388+RP4) community
	-303.03	0.001 ± 0.001	Plasmid R388 in 3-species (B, R, Y) 3-plasmid (R6K+R388+RP4) community
	-833.33	0.26 ± 0.00	Plasmid RP4 in 3-species (B, R, Y) 3-plasmid (R6K+R388+RP4) community

*The plasmid abundance data were extracted from Fig. 2, 3, and 4 of this referred paper. Data of 'Simple communities without linoleic acid and phenothiazine' were from Fig. 2c, Fig. 2d, and Fig. 3b. Data of 'Simple communities with linoleic acid and phenothiazine' were from Fig. 4c, Fig. 4d, and Fig. 4e. Data of 'Simple communities with antibiotic treatment' were from Fig. 2c and Fig. 3b. Data of 'Complex communities' were from Fig. 3a, Fig. 3c and Fig. 3d.

3.3 Compensatory mutations improve general permissiveness to antibiotic resistance plasmids

In this work, Loftie-Eaton *et al*. evolved *Pseudomonas sp.H2*, which carries multidrug resistance plasmid RP4, and determined how the capability of plasmid persistence changed with generations.

To verify that the change of plasmid persistence during the evolution was governed by a decrease in the ω value, we calculated the plasmid persistence potential of the ancestral pair (G0) as well as the 600-generation pair (G600), based on the parameter estimations provided by the authors (Supplementary Table 9). The δ factor quantifies the difference between the growth rates of plasmid-free cells (μ_0) and plasmid-carrying cells (μ_1) by $\delta = log_2(\mu_1/\mu_0)$. Therefore, the λ value in our model can be calculated as $\lambda = 2^{-\delta} - 1$. We estimated the dilution rate to be $0.0375\ h^{-1}$, and normalized the conjugation efficiency with 10^9. The death rate is much smaller than the growth rate, thus the term $\frac{\mu}{\mu-d}$ was approximated as 1 in our calculation. The ω values

and the corresponding relative abundance of plasmid RP4 are summarized in Supplementary Table 10.

Supplementary Table 9 Original data of plasmid RP4 relative abundance from 'Compensatory mutations improve general permissiveness to antibiotic resistance plasmids'

			Replicate 1	Replicate 2	Replicate 3
Ancestral pair (G0)	Population A		3/52	8/52	6/52
	Population B		4/52	4/52	0/52
	Population C		1/52	1/52	0/52
600th generation (G600)	Population A	Clone A1	52/52	52/52	52/52
		Clone A2	49/52	52/52	52/52
		Clone A3	52/52	51/52	51/52
	Population B	Clone B1	52/52	52/52	52/52
		Clone B2	51/52	50/52	52/52
		Clone B3	52/52	52/52	52/52
	Population C	Clone C1	50/52	50/52	50/52
		Clone C2	44/52	49/52	47/52
		Clone C3	49/52	43/52	40/52

*The plasmid abundance data, which were visualized in Fig. 1 of this referred paper, were obtained from DRYAD (https://datadryad.org/bitstream/handle/10255/dryad.147859/ResultsSection2.1.csv?sequence=1)

Supplementary Table 10 The parameter estimations, plasmid RP4 persistence potential and the corresponding plasmid relative abundance in 'Compensatory mutations improve general permissiveness to antibiotic resistance plasmids'

			δ	κ	η	ω	f
			Plasmid effect on fitness	Plasmid segregation loss rate	Plasmid conjugation efficiency	Plasmid persistence potential	Plasmid relative abundance
Ancestral pair (G0)	Population A		-6.59 %	$10^{-7.21}$	$10^{-12.85}$	0.084	0.11 ± 0.05
	Population B		-7.75 %	$10^{-6.51}$	$10^{-12.72}$	0.097	0.05 ± 0.04
	Population C		-7.91 %	$10^{-6.31}$	$10^{-12.80}$	0.079	0.01 ± 0.01
600th generation (G600)	Population A	Clone A1	2.48 %	$10^{-3.32}$	$10^{-13.54}$	-0.17	1 ± 0.00
		Clone A2	2.79 %	$10^{-3.38}$	$10^{-12.87}$	-0.43	0.98 ± 0.03
		Clone A3	2.56 %	$10^{-3.89}$	$10^{-12.92}$	-0.22	0.99 ± 0.01

	Population B	Clone B1	11.09 %	$10^{-3.70}$	$10^{-12.14}$	-0.26	1 ± 0.00
		Clone B2	6.43 %	$10^{-3.61}$	$10^{-12.95}$	-0.077	0.98 ± 0.02
		Clone B3	8.37 %	$10^{-4.08}$	$10^{-12.04}$	-0.42	1 ± 0.00
	Population C	Clone C1	10.16 %	$10^{-2.87}$	$10^{-12.69}$	-0.15	0.96 ± 0.00
		Clone C2	12.40 %	$10^{-2.65}$	$10^{-13.41}$	-0.035	0.90 ± 0.05
		Clone C3	8.45 %	$10^{-2.84}$	$10^{-12.92}$	-0.15	0.85 ± 0.09

*The plasmid effects on fitness (δ) were extracted from Fig. 3a of this referred paper. The plasmid segregation loss rates (κ) were extracted from Fig. 3b of this referred paper. The plasmid conjugation efficiencies (η) were extracted from Supplementary Fig. 2 of this referred paper. We changed the letters of each parameter to be consistent with our model notations.

3.4 Positive selection inhibits gene mobilization and transfer in soil bacterial communities

Hall JP *et al.* conducted a coculture experiment using *Pseudomonas fluorescens*, *Pseudomonas putica*, and plasmid pQBR57. We focused only on the data after transfer 29. We calculated the plasmid relative abundance from their original data (Supplementary Table 11). The parameter values were measured in their previous study[6]. Notably, in Fig. 2 of their main text, they also showed the results of short-term dynamics (transfer 0 to 5) of plasmid pQBR57 in *Pseudomonas fluorescens* cultured alone. However, we did not consider these results because at the end of the experiments (transfer 5), the population was far from the steady state.

Supplementary Table 11 The plasmid pQBR57 persistence potential and the corresponding relative abundance in 'Positive selection inhibits gene mobilization and transfer in soil bacterial communities'

	Plasmid persistence potential	Plasmid abundance
Transfer 29	1.39 ± 0.08	0.96875
Transfer 35	1.41 ± 0.09	0.96875
Transfer 41	1.39 ± 0.05	0.90625
Transfer 47	1.42 ± 0.06	0.93750
Transfer 53	1.34 ± 0.10	0.81250
Transfer 59	1.38 ± 0.06	0.96875
Transfer 65	1.13 ± 0.13	0.34375

*The plasmid persistence potentials were calculated from kinetic parameters given in our Supplementary Table 5. The plasmid abundance data, which were visualized in Fig. 3b of this referred paper, was obtained from DRYAD

(https://datadryad.org/bitstream/handle/10255/dryad.148630/S4_Tn5042_PF_Data.csv?sequence=1).

3.5 Parallel compensatory evolution stabilizes plasmids across the parasitism-mutualism continuum

Harrison E. *et al.*, investigated the compensatory evolution across the parasitism-mutualism continuum. They established 36 populations of bacterium *Pseudomonas fluorescens* SBW25 carrying plasmid QBR103, which encodes mercury resistance, and propagated the populations by serial transfer under six mercury concentrations ($0, 8, 16, 24, 32,$ and 40 μM $HgCl_2$). They analyzed the fitness of the ancestral and evolved generations in each environment as well as the conjugation rates. These data, combined with the plasmid abundance at the end of the transfers in each environment, are summarized in Supplementary Table 12.

The fitness value α of the evolved host-plasmid pair relative to the plasmid-free cells can be calculated by $\alpha = \alpha_A \alpha_E$, where α_A is the fitness of the ancestral pair relative to the plasmid-free cells and α_E is the fitness of the evolved pair relative to the ancestors. The values of α_E are summarized in Supplementary Table 12. However, the author did not directly provide the measured values of α_A under the six $HgCl_2$ concentrations. Instead, they provided a linear correlation between α_A and $HgCl_2$ concentrations with the unit of μM : $\alpha_A \approx 0.0371 \times [HgCl_2] + 0.3019$ (Supplementary Fig. 2 of their article). In this way, we were able to calculate the α values in the six cases. The λ values in our model can then be calculated by $\lambda = \alpha^{-1} - 1$. We estimated the dilution rate D of the transfers to be 0.0125. To become a dimensionless quantity, the conjugation efficiencies are required to be normalized with the maximum carrying capacity N_m. However, the authors did not provide this value in the article. Using similar experimental settings, Hall JP estimated the maximum carrying capacity of *Pseudomonas fluorescens* to be 6.01×10^8, as is shown in Supplementary Table 5, and we used this estimation in this analysis. We also used Hall's estimation of segregation rate $\kappa \approx 1 \times 10^{-4} h^{-1}$. The dilution rate is much smaller than the growth rate, thus the plasmid persistence potential can be approximated by $\omega \approx \bar{\eta} / \left(D + \bar{\kappa} - \frac{D}{1+\bar{\lambda}} \right)$. Our estimates of persistence potential and the corresponding plasmid abundance are summarized in Supplementary Table 12.

Supplementary Table 12 Kinetic parameters, persistence potential and plasmid abundance of plasmid pQBR103 in 'Parallel compensatory evolution stabilizes plasmids across the parasitism-mutualism continuum'

HgCl_2 concentration (μM)	Populations	α_E: Evolved fitness relative to ancestors	ln(conjugation rate)	Plasmid persistence potential	Plasmid relative abundance
0	A	1.30	-14.92	-1.05×10^4	0.701
	B	1.20	-15.77	-3.87×10^3	0.601
	C	1.51	-15.80	-5.55×10^3	0.403
	D	0.97	-15.97	-2.33×10^3	0.252
	E	1.27	-17.03	-1.21×10^3	0.094
	F	1.44	-16.90	-1.70×10^3	0.003
8	A	1.80	-17.44	1.61×10^4	1
	B	1.39	-17.94	-4.02×10^3	1
	C	1.22	-15.30	-3.05×10^4	1
	D	1.17	-15.34	-2.53×10^4	1
	E	1.08	-15.01	-2.71×10^4	1
	F	1.38	-16.63	-1.43×10^4	0.201
16	A	1.09	-14.07	-2.44×10^6	1
	B	1.15	-15.82	1.85×10^5	1
	C	1.33	-15.69	4.30×10^4	1
	D	1.57	-15.86	2.09×10^4	1
	E	1.33	-13.90	2.62×10^5	1
	F	1.41	-17.61	5.05×10^3	1
24	A	1.20	-15.41	3.16×10^4	0.901
	B	1.38	-15.37	2.54×10^4	1
	C	1.22	-15.49	2.77×10^4	1
	D	1.33	-15.97	1.47×10^4	1
	E	1.19	-16.60	9.71×10^3	1
	F	1.46	-17.75	2.17×10^3	1
32	A	1.35	-14.58	4.40×10^4	1
	B	1.40	-14.68	3.84×10^4	1
	C	1.23	-15.92	1.27×10^4	1
	D	1.11	-16.49	8.22×10^3	1
	E	1.29	-17.62	2.20×10^3	1
	F	1.39	-17.93	1.50×10^3	1
40	A	2.07	-15.07	1.86×10^4	1

	B	1.61	-15.45	1.42×10^4	1
	C	1.21	-16.51	5.96×10^3	1
	D	1.37	-16.82	3.99×10^3	1
	E	1.22	-16.77	4.54×10^3	1
	F	1.68	-17.48	1.83×10^3	1

*The α_E values were extracted from Fig. 1a of this referred article. We changed the letters of the parameter to fit the denotations of in our model. The conjugation rates were extracted from Fig. 2b of this referred article. The fitness of the ancestral pair relative to the plasmid-free cells were extracted from Supplementary Fig. 1, and the data of plasmid abundance were extracted from Supplementary Fig. 2 of this referred article.

3.6 Variable plasmid fitness effects and mobile genetic element dynamics across *Pseudomonas* species

Kottara A *et al*. tracked the dynamics of a large conjugative plasmid, pQBR103 (mercury-resistant), across five diverse *Pseudomonas* species (*P. fluorescens*, *P. savastanoi*, *P. stutzeri*, *P. aeruginosa*, and *P. putida*) in environments with and without mercury selection. They measured the fitness of the *Pseudomonas* species carrying the plasmid as well as the long-term frequencies of Hg^R phenotypes with or without 50 μM mercury. The pQBR103 conjugation efficiency of *P. fluorescens* can be obtained as $e^{-14.66}$ from their previous study[9]. The conjugation efficiencies of the other four species were lacking. Therefore, we only considered the data of *P. fluorescens*.

The λ value in our model was calculated from the selection rate γ through $\lambda = 2^{-\gamma} - 1$. We normalized the conjugation efficiency of *P. fluorescens* with respect to the carrying capacity 6.01×10^8 (Supplementary Table 5). We estimated the plasmid segregation rate κ to be 1×10^{-4} h^{-1} and the cellular death rate to be $0.009\ h^{-1}$ (Supplementary Table 5). When calculating the persistence potential $\omega = \frac{\bar{\eta}}{\frac{\bar{\mu}}{\bar{\mu}-\sigma d}\left(d+\bar{\kappa}-\frac{d}{1+\bar{\lambda}}\right)}$, we approximated the term $\frac{\bar{\mu}}{\bar{\mu}-\sigma d}$ to be 1 since the growth rate was much greater than the death rate. Therefore, we obtained the plasmid persistence potential as $\bar{\eta}/\left(d+\bar{\kappa}-\frac{d}{1+\bar{\lambda}}\right)$. The ω values and the corresponding plasmid relative abundance data are summarized in Supplementary Table 13.

Supplementary Table 13 Kinetic parameters, persistence potential and relative abundance of plasmid pQBR103 in 'Variable plasmid fitness effects and mobile genetic element dynamics across *Pseudomonas* species'

	Description	Value
γ_0	The selection rate of P. Fluorescens carrying plasmid pQBR103 without mercury selection	-0.425
γ_{50}	The selection rate of P. Fluorescens carrying plasmid pQBR103 with 50 μM mercury selection	2.053
ω_0	Persistence potential of plasmid pQBR103 without mercury selection	1.08×10^5
ω_{50}	Persistence potential of plasmid pQBR103 with 50 μM mercury selection	-9.14×10^3
f_{60}^0	Plasmid pQBR103 abundance without selection at transfer 60	0.69 ± 0.17
f_{48}^0	Plasmid pQBR103 abundance without selection at transfer 48	0.75 ± 0.06
f_{36}^0	Plasmid pQBR103 abundance without selection at transfer 36	0.83 ± 0.09
f_{60}^{50}	Plasmid pQBR103 abundance with 50 μM mercury selection at transfer 60	0.75 ± 0.03
f_{48}^{50}	Plasmid pQBR103 abundance with 50 μM mercury selection at transfer 48	0.81 ± 0.08
f_{36}^{50}	Plasmid pQBR103 abundance with 50 μM mercury selection at transfer 36	0.82 ± 0.04

*The γ values were extracted from Fig. 1 of this referred article. The data of plasmid abundance were extracted from Fig. 2 of this referred article.

3.7 Amelioration of the cost of conjugative plasmid carriage in *Escherichia coli* K12

Dahlberg C *et al.* studied how the cost of conjugative plasmids carrying drug resistance evolved in batch cultures in the absence of antibiotic selection. They studied two plasmids, R1 and RP4, both of which carried multiple drug resistance genes and imposed an initial fitness cost on the host *Escherichia coli*. They subjected the plasmid-carrying bacteria to 1100 generations of evolution. They then measured the changes in fitness costs as well as the conjugation efficiencies.

The plasmid-abundance data of RP4 were not presented in the paper, so we only considered the plasmid R1. The fitness costs, conjugation rates, and plasmid abundance of three evolved R1 populations are shown in Supplementary Table 14. The λ factors can be obtained from the fitness cost α through $\lambda = \alpha - 1$. We normalized the conjugation efficiency using the maximum carrying capacity $N_m = 8 \times 10^9$. We estimated the segregation rate to be 0.001, and the dilution rate D to be 0.025. We also assumed the growth rates to be much larger than the dilution rate, so that the factor $\frac{\mu}{\mu-D}$ was approximated as 1. The values of plasmid persistence potential ω are also summarized in Supplementary Table 14.

Supplementary Table 14 Kinetic parameters, persistence potential and relative abundance of plasmid R1 in 'Amelioration of the cost of conjugative plasmid carriage in *Eschericha coli* K12'

	Population 1	Population 2	Population 3
Fitness cost α	1.286	1.303	1.309
Conjugation efficiency η	2.25×10^{-12}	1.14×10^{-12}	9.14×10^{-12}
Plasmid fraction	105/106	97/106	66/88
Plasmid persistence potential ω	2.74	1.34	10.59

*The α values were extracted from Table 2 of this referred article. Population 1, 2, 3 correspond to strain CD111, CD112, and CD113, respectively. The conjugation rates were obtained from Table 4, and the plasmid fractions were obtained from Table 3.

3.8 The IncI1 plasmid carrying the bla$_{\text{CTX-M-1}}$ gene persists in in vitro culture of an *Escherichia coli* strain from broilers

Fischer EA *et al.* studied the conjugation dynamics of the lncl1 plasmid carrying *bla$_{CTX\text{-}M\text{-}1}$* gene in a batch culture. They quantified the population dynamics of three *E. coli* populations (donors, recipients, and transconjugants) in two mixed-culture experiments. In the first experiment, donor cells were mixed with recipient cells and the mixture was incubated for 24 hours without dilution. To determine the cell densities of donors, recipients, and transconjugants, the samples were taken out for colony counts at 0, 3, 6, 16, 19, and 24 h after the start of experiments. In the second experiment, transconjugants and recipients were mixed, and the culture were passaged every 24 hours or 48 hours with a dilution ratio of 1:10,000. The cultures were passaged over a period of three months. The cell densities of recipients and transconjugants were measured through plating and colony counts. The plasmid abundance data and the estimations of kinetic parameters are summarized in Supplementary Table 15.

In the first experiment, there was no dilution ($D = 0$). Therefore, the plasmid persistence potential ω can be obtained as $\omega = \frac{\bar{\eta}}{\kappa}$. The plasmid loss rate was provided as $0.0008{\sim}0.0036$. We used the median value 0.0022 as the estimation of κ. We calculated $\bar{\eta}$ through $\bar{\eta} = \frac{(\eta_D N_m)N_D + (\eta_T N_m)N_T}{N_D + N_R + N_T}$, where N_D, N_R, and N_T are the cell densities of donors, recipients, and transconjugants, respectively. η_D and η_T are the conjugation rates of donors and transconjugants,

respectively. Here we normalized the conjugation rates with respect to the maximum carrying capacity N_m.

In the second experiment, the conjugation efficiency became $\eta = \eta_T N_m$. For the dilution period of 24 hours, we estimated the dilution rate $D = 0.05$, and for the dilution period of 48 hours, we estimated $D = 0.025$. The λ factor could be obtained through $\lambda = \frac{\mu_R}{\mu_D} - 1$, where μ_R and μ_D are the growth rates of donors and recipients, respectively. The ω values in both cases could then be calculated through $\omega = \frac{\eta}{\frac{\mu_R}{\mu_R - D}\left(D + \kappa - \frac{D}{1+\lambda}\right)}$. The ω values and the corresponding plasmid abundances are summarized in Supplementary Table 15.

As is shown in Supplementary Table 15, the plasmid abundances of the long-term experiments were higher than 1. This is due to the protocols the authored employed to measure plasmid abundance. The authors used selective plating to determine the relative abundance of plasmid-carrying cells. In the mixture of the plasmid-carrying cells (denoted as T) and plasmid-free cells (denoted as R), plasmid-free cells were ciprofloxacin-resistant while plasmid-carrying cells were resistant to both ciprofloxacin and cefotaxime. They first measured the total density of cells (R+T) using ciprofloxacin selection. Then, they measured the density of plasmid-carrying cells (T) using double selection (ciprofloxacin and cefotaxime). Ideally, the measured value of T should also be smaller than R+T. However, because (1) the fraction of R was very small; (2) the relative error of selective plating and colony counting was relatively big, the measured value of T sometimes exceeded the measured value of R+T, which is a result of experimental artifact. Therefore, the relative abundances of these two data points were higher than 1.

Supplementary Table 15 Kinetic parameters, persistence potential and relative abundance of IncI1 plasmid in 'The IncI1 plasmid carrying the *bla*$_{\text{CTX-M-1}}$ gene persists in *in vitro* culture of an *Escherichia coli* strain from broilers'

Description	Value
Growth rate of recipient cells μ_R	$2.04\ h^{-1}$
Growth rate of donor cells μ_D	$2.09\ h^{-1}$
Growth rate of transconjugant cells μ_T	$2.09\ h^{-1}$

Maximum carrying capacity of the mixture N_m			$9.33 \times 10^8\ cfu/ml$
Plasmid loss rate κ			$0.008\sim0.0036\ h^{-1}$
Conjugation efficiency of donor cells η_D			$2.4 \times 10^{-14}\ cfu^{-1}h^{-1}ml$
Conjugation efficiency of transconjugant cells η_T			$4.4 \times 10^{-10}\ cfu^{-1}h^{-1}$
Short-term experiments without dilution		Population density of donors N_D	4.02×10^8
		Population density of recipients N_R	6.19×10^8
		Population density of transconjugants N_T	6.19×10^7
		Plasmid persistence potential ω	117.34
		Plasmid abundance f	0.43
Long-term experiments with dilution	Dilution every 24 hours	Plasmid persistence potential ω	410.93
		Plasmid abundance f	1.03 ± 0.28
	Dilution every 48 hours	Plasmid persistence potential ω	255.47
		Plasmid abundance f	0.94 ± 0.28

*The values of μ_R, μ_D, μ_T were obtained from Table 1 of this referred paper. The value of N_m was obtained from Table 2. The value of κ was obtained from the main text. The values of η_D and η_T were obtained from Table 3. The population density data of short-term experiments were extracted from Fig. 2 of this referred paper. The plasmid abundance data of long-term experiments were extracted from Fig. 3. We changed the letters of each parameters to be consistent with our model notations.

3.9 Survival and evolution of a large multidrug resistance plasmid in new clinical bacterial hosts

Porse A *et al.* investigated the survival and evolution of a large multidrug resistance plasmid, pKp33, in three clinical bacterial hosts: Ec37, Ec38 and Kp08. They evolved the host-plasmid pairs for ~280 generations and then measured the plasmid stability of both naïve and evolved host-plasmid pairs. During the measurements, the plasmid dynamics in evolved host-plasmid pairs did not reach steady states, thus we only considered the dynamics of naïve plasmid-host pairs. The values of kinetic parameters are summarized in Supplementary Table 16.

During the experiments, they adopted a daily dilution ratio of 1:150. Therefore, we estimated the dilution rate D to be 0.0272. We approximated the plasmid persistence potential as $\omega = \eta / \left(D + \kappa - \frac{D}{1+\lambda}\right)$, since the dilution rate is much smaller than the growth rate. λ values can be obtained from ρ through $\lambda = \frac{1}{1-\rho} - 1$. We also normalized the conjugation efficiency with the

maximum carrying capacity $N_m = 1 \times 10^9$. The plasmid persistence potentials and the corresponding plasmid abundance data are summarized in Supplementary Table 16.

Supplementary Table 16 Kinetic parameters, plasmid persistence potential and plasmid relative abundance data in 'Survival and evolution of a large multidrug resistance plasmid in new clinical bacterial hosts'

	Ec37/pKP33	Ec38/pKP33	Kp08/pKP33
Conjugation rate η	5.03×10^{-14}	4.21×10^{-13}	7.17×10^{-14}
Segregation rate κ	0.0147	0.0008	0.0060
Plasmid cost ρ	8.3%	14.0%	4.6%
Plasmid persistence potential ω	0.003	0.091	0.99
Plasmid relative abundance	0	0	0

*The values of conjugation rates were obtained from Supplementary Table 4 of this referred paper. The values of segregation rates and plasmid costs were obtained from Supplementary Table 5 of the paper. The plasmid abundance data of were extracted from Fig. 4. We changed the letters of each parameter to be consistent with our model notations.

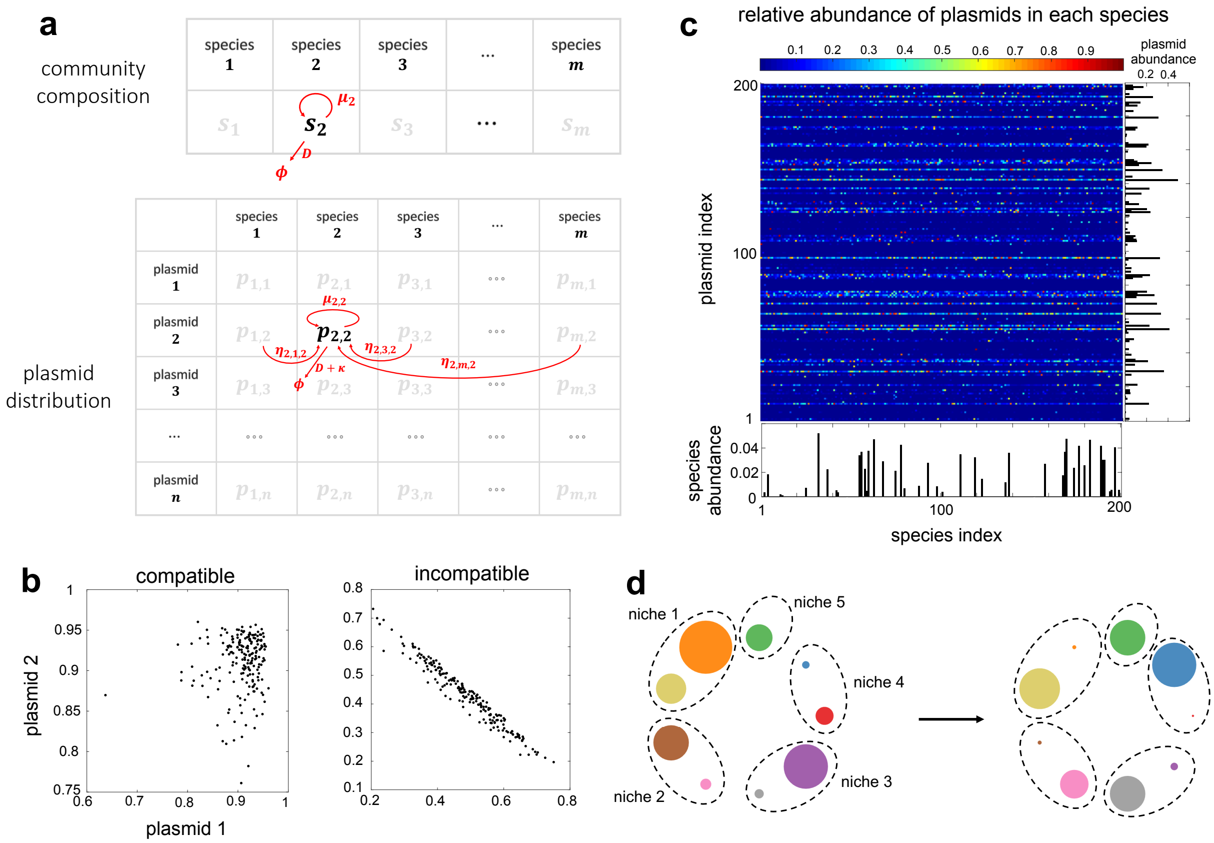

Supplementary Fig. 1 Plasmid-centric framework (PCF) and its application in predicting microbial community dynamics.

a Dynamic processes in PCF. The variables in PCF include the species abundance (s) and plasmid abundance in each species (p). Four processes are described in this framework: cell division (μ), dilution (D), plasmid horizontal transfer (η), and plasmid loss (κ).

b PCF can be applied to incompatible plasmids. A community of 200 species and 2 plasmids, compatible or incompatible with each other, are shown as an example. In each species, the relative abundance of the plasmids, calculated as the fraction of the plasmid-carrying cells, are calculated and plotted. The simulation was carried out with the randomized parameters within the following ranges: $0.4 \le \mu \le 0.8$, $0.001 \le D \le 0.005$, $0 \le \kappa \le 0.002$, $0 \le \lambda \le 0.2$ and $0 \le \eta \le 0.04$. When the two plasmids are incompatible, their relative abundances in each species, referred to as x, y, exhibit a strong negative correlation and are constrained by $x + y < 1$, suggesting that these two plasmids cannot coexist stably in the same host cell.

c PCF is capable of predicting the steady-state composition, plasmid abundance, and distribution patterns of huge communities. A community with 200 species and 200 plasmids is shown as an example here. All parameters are randomized. The relative abundance of plasmid j in species i was calculated as the fraction of species i cells that contains plasmid j relative to the total number of species i cells.

d In testing the persistence potential, the species were distributed into multiple niches. Each niche had its own carrying capacity, and different species within the same niche competed with each other.

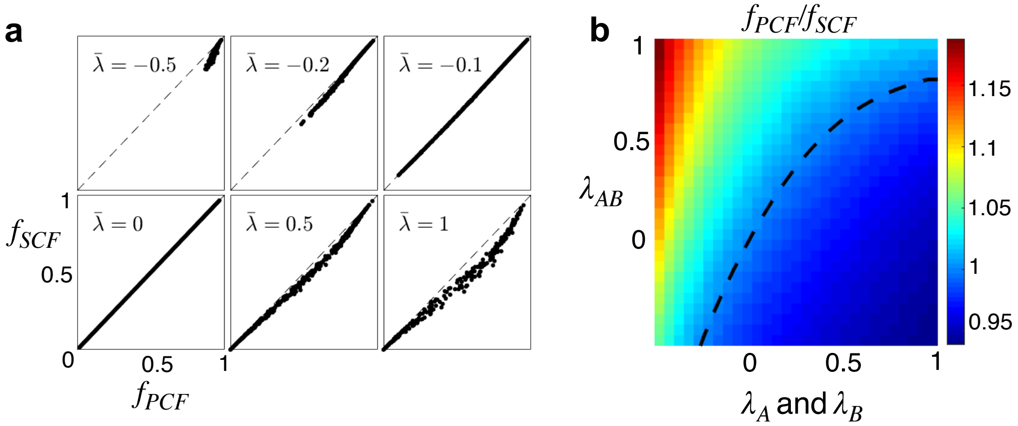

Supplementary Fig. 2 Systematic discrepancy between PCF and SCF.

a For single-PCF communities, the discrepancy increases with higher burden or benefit. One thousand numerical simulations were performed in each of the six tests, with all the parameters in PCF and SCF randomized in the range of $1 \leq m \leq 50$, $1.6 \times 10^{-3} \leq \kappa \leq 2.4 \times 10^{-3}$, $1.6 \times 10^{-2} \leq D \leq 2.4 \times 10^{-2}$, $0 \leq \eta \leq 0.02$, and $0.3 \leq \mu \leq 0.8$. The steady-state abundance of the plasmid, defined as the fraction of plasmid-carrying cells in the total population, was calculated as f_{PCF} or f_{SCF}.

b For two-plasmid communities, the discrepancy depends on their individual fitness costs (λ_A and λ_B) as well as their combined effect (λ_{AB}). The parameters used in the simulations are $\kappa = 0.001$, $D = 0.005$, $\mu = 0.3$, $\eta = 0.01$. The ratio of f_{MCF} to f_{SCF} was calculated as the output. The conditions under which PCF and SCF generate the same plasmid abundance ($\frac{f_{MCF}}{f_{SCF}} = 1$) are shown in black dashed curve.

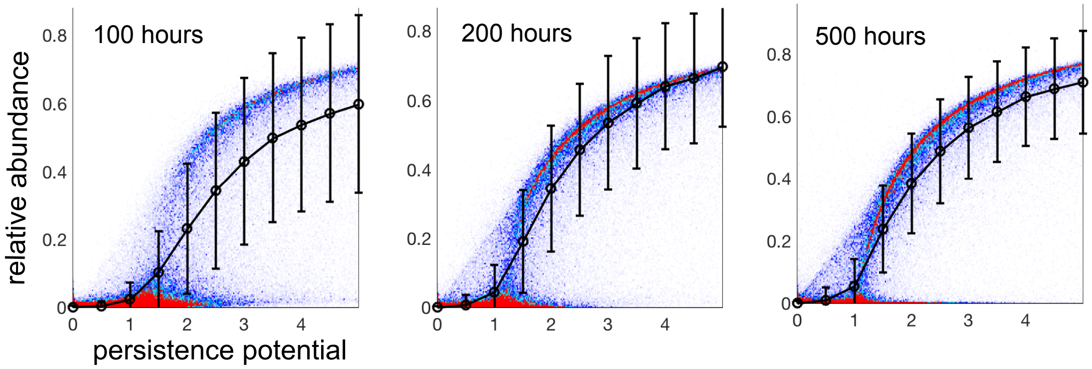

Supplementary Fig. 3 ω predicts temporal abundance of plasmids before the communities reach steady states. 50,000 simulations were performed with 1-10 populations, 1-10 plasmids, and randomized parameters in the range of $0.1 \leq \mu \leq 0.8, 0.01 \leq D \leq 0.05$, $0 \leq \kappa \leq 0.1$, $0 \leq \lambda \leq 0.2$. For each simulation, the communities were assembled into a random number of niches. Within each niche, populations compete with each other. Each simulation was initialized with random abundances of populations and plasmids. The persistence potential ω of each plasmid and its relative abundance in the entire community at different time points were then calculated. The ω range is divided into multiples bins with widths of 0.5. Data are presented as the mean values +/- standard deviations of all the plasmid abundances within each bin. Each bin contains 812 to 100321 independent replicates.

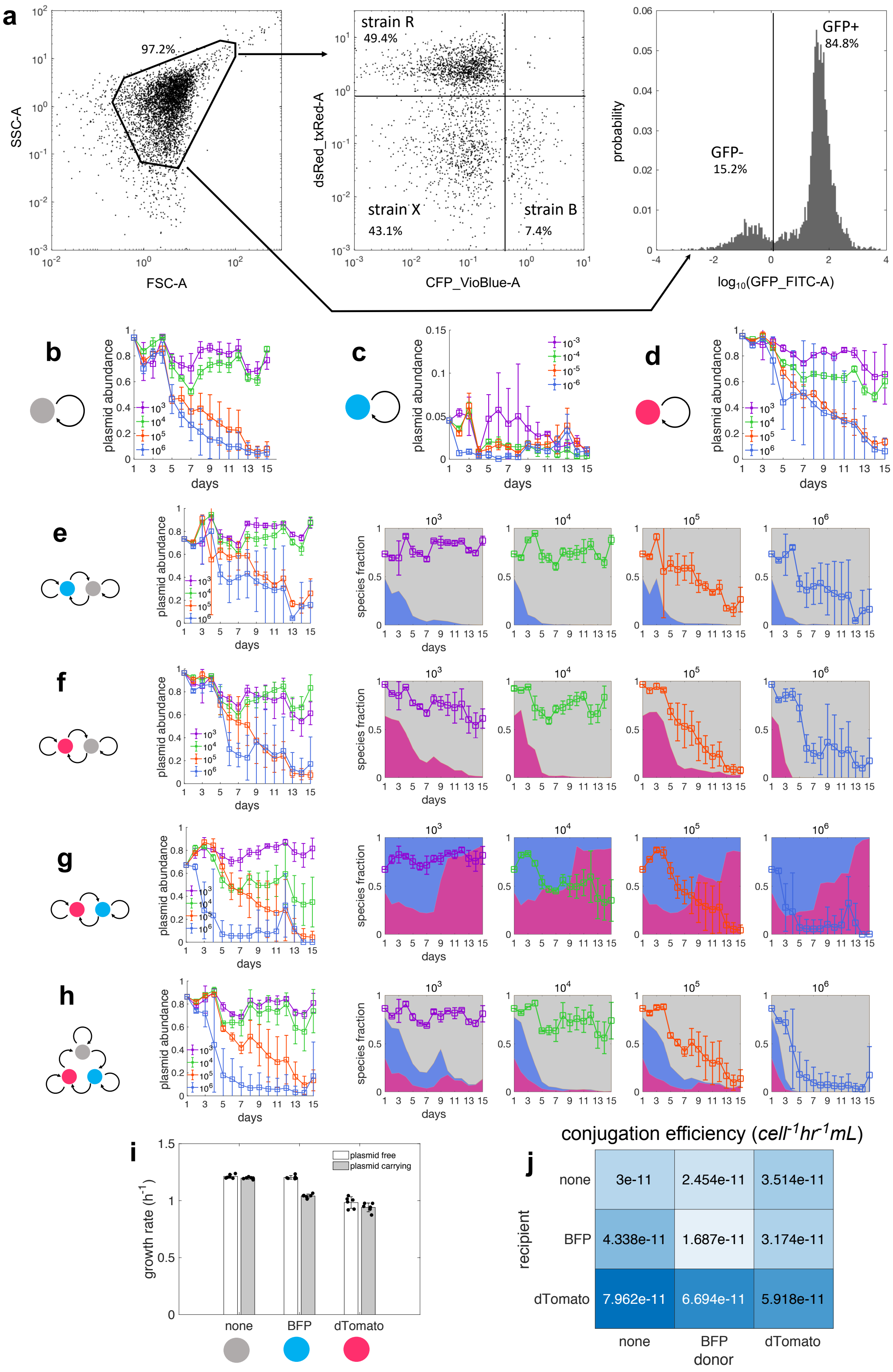
a
97.2%
SSC-A
FSC-A
strain R
49.4%
strain X
43.1%
strain B
7.4%
dsRed_txRed-A
CFP_VioBlue-A
GFP+
84.8%
GFP-
15.2%
probability
$\log_{10}$(GFP_FITC-A)
b
c
d
plasmid abundance
days
10^3
10^4
10^5
10^6
10^{-3}
10^{-4}
10^{-5}
10^{-6}
e
f
g
h
species fraction
i
growth rate (h^{-1})
plasmid free
plasmid carrying
none
BFP
dTomato
j
conjugation efficiency ($cell^{-1}hr^{-1}mL$)
recipient
donor
none 3e-11 2.454e-11 3.514e-11
BFP 4.338e-11 1.687e-11 3.174e-11
dTomato 7.962e-11 6.694e-11 5.918e-11
none BFP dTomato

Supplementary Fig. 4 Long-term experiments and parameter estimations of the seven communities.

a Determination of community composition and plasmid abundance using flow cytometry. CFP_VioBlue channel was used to detect BFP expressed by strain B, and dsRed_txRed channel was used to detect dTomato expressed by strain R. The cells with low BFP and dTomato signals represent strain X. The plasmid-carrying cells, which express plasmid-borne GFP, were detected by channel GFP_FITC.

b-h The long-term dynamics of population composition and plasmid abundance in seven communities measured by flow cytometry. The filled circles stand for three *E. coli* strains. The strain expressing BFP chromosomally is shown in blue, the strain expressing dTomato is shown in red, and the non-fluorescent strain is shown in gray. The arrows shown in black represent the conjugation of the GFP-expressing plasmid K. Three communities each consisted of single populations (**b-d**), three communities each consisted of two members (**e-g**), and one consisted of all three members (**h**). Four daily dilution ratios (10^3, 10^4, 10^5m and 10^6) were applied to each community. The plasmid dynamics are shown in line plots. Data are presented as the mean value +/- standard deviation of three replicates. The composition dynamics are shown in colored areas.

i The growth rates of plasmid-free and plasmid-carrying cells of the three strains. The growth rates were calculated as the rate constants of the exponential phase. Data are presented as the mean value +/- standard deviation of six replicates.

j The conjugation efficiencies among the three strains. Nine donor-recipient pairs were obtained, and the values of their conjugation rates are shown in the color map.

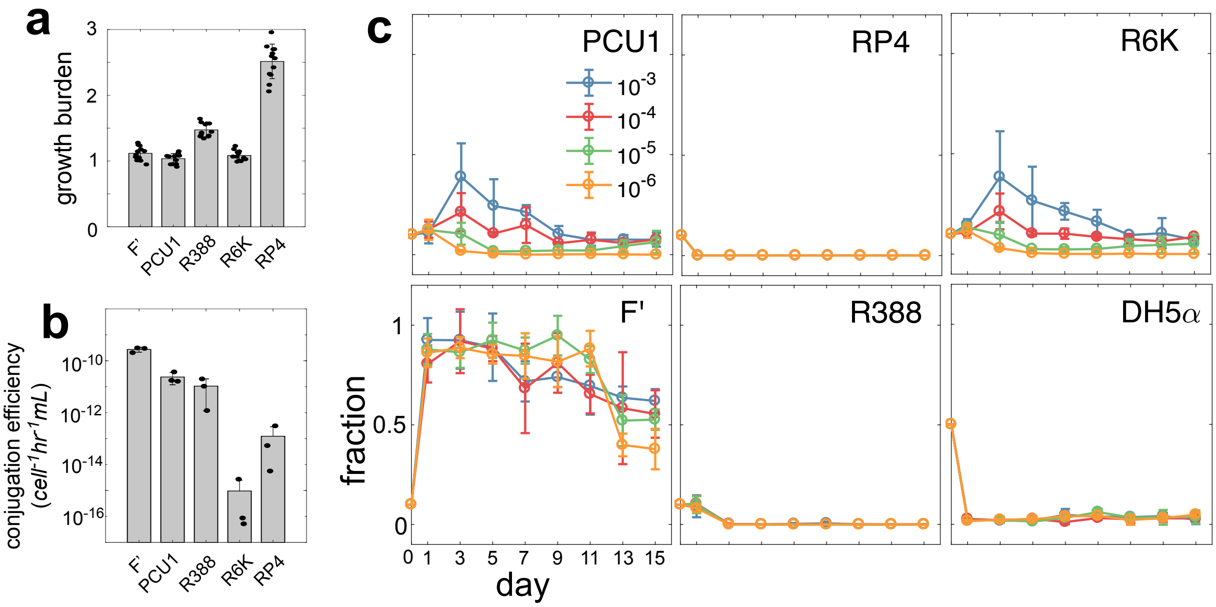

Supplementary Fig. 5 Long-term experiments and parameter estimations of the five-plasmid communities.

a The growth burden of the five conjugative plasmids in the strain MG1655. Here, the growth burden was quantified as the ratio between the growth rates of plasmid-free cells and plasmid-carrying cells. Data are presented as the mean values +/- standard deviations of twelve biologically independent replicates.

b The conjugation efficiencies of the five conjugative plasmids between MG1655 cells. Data are presented as the mean values +/- standard deviations of three biologically independent replicates.

c The long-term dynamics of population composition and plasmid abundance in the five-plasmid communities measured by plating. Four daily dilution ratios (10^3, 10^4, 10^5, and 10^6) were applied to each community. The relative abundances of the plasmids (F', PCU1, R388, R6K, and RP4) and the strain DH5α are shown in line plots. Data are presented as the mean values +/- standard deviations of three biologically independent replicates.

References

1 Lopatkin, A. J. *et al.* Persistence and reversal of plasmid-mediated antibiotic resistance. *Nature communications* **8**, 1689 (2017).

2 Lenski, R. E. & Levin, B. R. Constraints on the coevolution of bacteria and virulent phage: a model, some experiments, and predictions for natural communities. *The American Naturalist* **125**, 585-602 (1985).

3 Levin, B. R., Stewart, F. M. & Chao, L. Resource-limited growth, competition, and predation: a model and experimental studies with bacteria and bacteriophage. *The American Naturalist* **111**, 3-24 (1977).

4 Condit, R., Stewart, F. M. & Levin, B. R. The population biology of bacterial transposons: a priori conditions for maintenance as parasitic DNA. *The American Naturalist* **132**, 129-147 (1988).

5 Condit, R. The evolution of transposable elements: conditions for establishment in bacterial populations. *Evolution* **44**, 347-359 (1990).

6 Hall, J. P., Wood, A. J., Harrison, E. & Brockhurst, M. A. Source–sink plasmid transfer dynamics maintain gene mobility in soil bacterial communities. *Proceedings of the National Academy of Sciences* **113**, 8260-8265 (2016).

7 Loftie-Eaton, W. *et al.* Compensatory mutations improve general permissiveness to antibiotic resistance plasmids. *Nature ecology & evolution* **1**, 1354 (2017).

8 Hall, J. P., Williams, D., Paterson, S., Harrison, E. & Brockhurst, M. A. Positive selection inhibits gene mobilization and transfer in soil bacterial communities. *Nature ecology & evolution* **1**, 1348 (2017).

9 Harrison, E., Guymer, D., Spiers, A. J., Paterson, S. & Brockhurst, M. A. Parallel compensatory evolution stabilizes plasmids across the parasitism-mutualism continuum. *Current Biology* **25**, 2034-2039 (2015).

10 Kottara, A., Hall, J. P., Harrison, E. & Brockhurst, M. A. Variable plasmid fitness effects and mobile genetic element dynamics across Pseudomonas species. *FEMS microbiology ecology* **94**, fix172 (2017).

11 Dahlberg, C. & Chao, L. Amelioration of the cost of conjugative plasmid carriage in Eschericha coli K12. *Genetics* **165**, 1641-1649 (2003).

12 Fischer, E. A. *et al.* The IncI1 plasmid carrying the bla CTX-M-1 gene persists in in vitro culture of a Escherichia coli strain from broilers. *BMC microbiology* **14**, 77 (2014).

13 Porse, A., Schønning, K., Munck, C. & Sommer, M. O. Survival and evolution of a large multidrug resistance plasmid in new clinical bacterial hosts. *Molecular biology and evolution* **33**, 2860-2873 (2016).
