# Supplementary material for: The persistence potential of transferable plasmids
Source: Nat Commun. 2020 Nov 4;11:5589. doi: 10.1038/s41467-020-19368-7 (PMC7642394; doi:10.1038/s41467-020-19368-7)
Supplement: Supplementary file 3 — Reporting Summary [file 41467_2020_19368_MOESM3_ESM.pdf]

nature research

Corresponding author(s): Lingchong You

Last updated by author(s): Sep 22, 2020

Reporting Summary

Nature Research wishes to improve the reproducibility of the work that we publish. This form provides structure for consistency and transparency in reporting. For further information on Nature Research policies, see our Editorial Policies and the Editorial Policy Checklist.

Statistics

For all statistical analyses, confirm that the following items are present in the figure legend, table legend, main text, or Methods section.

n/a	Confirmed	
☐	☒	The exact sample size (*n*) for each experimental group/condition, given as a discrete number and unit of measurement
☐	☒	A statement on whether measurements were taken from distinct samples or whether the same sample was measured repeatedly
☒	☐	The statistical test(s) used AND whether they are one- or two-sided<br>*Only common tests should be described solely by name; describe more complex techniques in the Methods section.*
☒	☐	A description of all covariates tested
☐	☒	A description of any assumptions or corrections, such as tests of normality and adjustment for multiple comparisons
☐	☒	A full description of the statistical parameters including central tendency (e.g. means) or other basic estimates (e.g. regression coefficient) AND variation (e.g. standard deviation) or associated estimates of uncertainty (e.g. confidence intervals)
☒	☐	For null hypothesis testing, the test statistic (e.g. *F*, *t*, *r*) with confidence intervals, effect sizes, degrees of freedom and *P* value noted<br>*Give P values as exact values whenever suitable.*
☒	☐	For Bayesian analysis, information on the choice of priors and Markov chain Monte Carlo settings
☐	☒	For hierarchical and complex designs, identification of the appropriate level for tests and full reporting of outcomes
☒	☐	Estimates of effect sizes (e.g. Cohen's *d*, Pearson's *r*), indicating how they were calculated

Our web collection on statistics for biologists contains articles on many of the points above.

Software and code

Policy information about availability of computer code

Data collection	FlowJo (version 10.5.3) was used to collect the flow cytometry data.
Data analysis	FlowJo (version 10.5.3) was used to analyze the flow cytometry data.<br>MATLAB (R2017a) was used to calculate the parameter values and to perform the numerical simulations. The Matlab codes to generate the simulation results are available at the Github repository (https://github.com/youlab/PlasmidPersistencePotential_TengWang).

For manuscripts utilizing custom algorithms or software that are central to the research but not yet described in published literature, software must be made available to editors and reviewers. We strongly encourage code deposition in a community repository (e.g. GitHub). See the Nature Research guidelines for submitting code & software for further information.

Data

Policy information about availability of data

All manuscripts must include a data availability statement. This statement should provide the following information, where applicable:

- Accession codes, unique identifiers, or web links for publicly available datasets
- A list of figures that have associated raw data
- A description of any restrictions on data availability

The authors declare that all data of this study are available within the manuscript and its supplementary file. The summary of the literature data is provided in supplementary tables S3 to S16. The simulation data associated with figure 1, figure 3, and supplementary figures 1 to 3 are generated by the matlab codes that are available at the Github repository (See Code Availability). Source data associated with Figure 4, supplementary figure S4 and S5 are provided with this paper. Any additional information is available upon request.

Field-specific reporting

Please select the one below that is the best fit for your research. If you are not sure, read the appropriate sections before making your selection.

- [x] Life sciences
- [ ] Behavioural & social sciences
- [ ] Ecological, evolutionary & environmental sciences

For a reference copy of the document with all sections, see nature.com/documents/nr-reporting-summary-flat.pdf

Life sciences study design

All studies must disclose on these points even when the disclosure is negative.

Sample size	For the temporal dynamics of the engineered communities, 3 biologically independent samples were measured. n=3 was used for the measurement of conjugation efficiency. For the growth rates and the plasmid burden, at least 6 biologically independent samples were measured. The sample sizes were chosen based on the scale of the project and consistency with other similar published studies. No sample-size calculation was performed. Our results suggested that the sample sizes we chose were sufficient to test the theoretical predictions.
Data exclusions	No data were excluded for the analysis
Replication	Each experiment and simulation were repeated at least three times with similar results, suggesting the robustness of our conclusions.
Randomization	In the experiments on the engineered communities, the samples were not randomized since position of samples in multi-well plates and order of flow cytometric acquisition was not expected to affect the conclusion.
Blinding	No group allocation was involved in this study. Knowledge of a samples identity did not affect the experimental conclusion.

Reporting for specific materials, systems and methods

We require information from authors about some types of materials, experimental systems and methods used in many studies. Here, indicate whether each material, system or method listed is relevant to your study. If you are not sure if a list item applies to your research, read the appropriate section before selecting a response.

Materials & experimental systems

n/a	Involved in the study	
x		Antibodies
x		Eukaryotic cell lines
x		Palaeontology and archaeology
x		Animals and other organisms
x		Human research participants
x		Clinical data
x		Dual use research of concern

Methods

n/a	Involved in the study	
x		ChIP-seq
	x	Flow cytometry
x		MRI-based neuroimaging

Flow Cytometry

Plots

Confirm that:

- [x] The axis labels state the marker and fluorochrome used (e.g. CD4-FITC).
- [x] The axis scales are clearly visible. Include numbers along axes only for bottom left plot of group (a 'group' is an analysis of identical markers).
- [x] All plots are contour plots with outliers or pseudocolor plots.
- [x] A numerical value for number of cells or percentage (with statistics) is provided.

Methodology

Sample preparation	From day 1 to day 15, the overnight cultures of E. coli (in M9 mdeia) were resuspended and diluted to 1: 1000 in 200 µL fresh M9 media before running through the flow.
Instrument	MACSQuant® VYB Analyzer
Software	FlowJo (version 10.5.3)

Cell population abundance	Cell sorting was not performed in this study. Only flow cytometry cell analysis was involved.
Gating strategy	First, the E.coli cells were gated based on FSC-A and SSC-A. Then the BFP+ and dTomato+ cells were gated within the population to determine the community composition. The population was then analyzed for GFP expression to determine the plasmid abundance. A representative example for the gating strategy was provided in supplementary figure S4a.

☒ Tick this box to confirm that a figure exemplifying the gating strategy is provided in the Supplementary Information.
